# Development and validation of a machine learning model for online predicting the risk of in heart failure: based on the routine blood test and their derived parameters

**DOI:** 10.3389/fcvm.2025.1539966

**Published:** 2025-03-17

**Authors:** Jianchen Pu, Yimin Yao, Xiaochun Wang

**Affiliations:** ^1^ Medical Laboratory, Nanxun Hospital of Traditional Chinese Medicine, Huzhou, China; ^2^ Medical Laboratory, The First Affiliated Hospital of Zhejiang Chinese Medical University (Zhejiang Provincial Hospital of Chinese Medicine), Hangzhou, China

**Keywords:** routine blood test, heart failure, machine learning, clinical research, Random Forest

## Abstract

**Background:**

Heart failure (HF), a core component of cardiovascular diseases, is characterized by high morbidity and mortality worldwide. By collecting and analyzing routine blood data, machine learning models were built to identify the patterns of changes in blood indicators related to HF.

**Methods:**

We conducted a statistical analysis of routine blood data from 226 patients who visited Zhejiang Provincial Hospital of Traditional Chinese Medicine (Hubin) between May 1, 2024, and June 30, 2024. The patients were divided into an experimental group (HF patients) and a normal control group. Additionally, 211 patients from the Qiantang and Xixi centers formed an independent external validation cohort. This study used both univariate and multivariate analyses to identify the risk factors associated with HF. Variables associated with HF were selected using LASSO regression analysis. In addition, eight different machine learning algorithms were applied for prediction, and the prediction performances of these algorithms were comprehensively evaluated using the receiver operating characteristic curve, area under the curve (AUC), calibration curve analysis, and decision curve analysis and confusion matrix.

**Conclusions:**

Using LASSO regression analysis, leukocyte, neutrophil, red blood cell, hemoglobin, platelet, and monocyte-to-lymphocyte ratios were identified as risk factors for HF. Among the evaluated models, the random forest model exhibited the best performance. In the validation cohort, the area under the curve (AUC) of the model was 0.948, while that of the test cohort was 1.000. The calibration curve revealed good agreement between the actual and predicted probabilities, whereas the decision curve showed the significant clinical application of the model. Additionally, the AUC of the model in the external independent test cohort was 0.945.

**Discussion:**

We used an online predictive tool to develop a predictive machine-learning model. The main purpose of this model was to predict the probability of developing HF in the future. This prediction can provide strong support and references for clinicians when making decisions. This online forecasting tool not only processes a large amount of data but also continuously optimizes and adjusts the accuracy of the model according to the latest medical research and clinical data. We hope to identify high-risk patients for early intervention to reduce the incidence of HF and improve their quality of life.

## 1 Introduction

With an increasing aging population, the incidence of HF is increasing annually, placing great pressure on the global medical system (1). Recently, there has been increasing interest in identifying biomarkers that contribute to the early detection, prognosis, and monitoring of cardiovascular diseases. One such biomarker is brain diuretic natriuretic peptide (Pro-BNP) (2–4). Pro-BNP is a precursor peptide synthesized and released from the ventricular myocardium in response to cardiac stress and strain. High pro-BNP levels are associated with various cardiac conditions such as HF, myocardial infarction, and atrial fibrillation. Measurement of pro-BNP has become an important tool for the diagnosis and prognosis of patients with HF in clinical application (5–8). However, the implementation of pro-BNP testing poses certain difficulties for primary healthcare institutions. Therefore, exploring early diagnostic markers and effective treatments for HF is crucial for improving patient prognosis, enhancing the quality of life, and reducing healthcare costs.

The CBC is a routine clinical test that reflects the status of the inflammatory response in the body. Recent research has found that certain indicators in complete blood cell count, such as white blood cell count, neutrophil count, and lymphocyte count, are closely related to the occurrence and development of HF (9). These indicators are not only simple, rapid, and economical but can also reflect the response status and degree of patients with HF, which has important reference value for early diagnosis.

Machine learning (ML) is a branch of artificial intelligence that focuses on how computers simulate or implement human learning processes to master new knowledge or skills and reorganize existing knowledge structures to continuously improve their performance (10). In recent years, machine learning has been increasingly used in healthcare, particularly for predicting HF (11). As simple and economical tests, routine blood tests show great potential for predicting HF (9). By collecting and analyzing large amounts of routine blood data, machine learning models can be constructed to identify patterns of change in blood indicators associated with HF. These models can be used to assess the risk of HF and provide strong support for clinical decision-making.

HF exhibits features of high morbidity and high mortality (12). By collecting and analyzing routine blood data, machine learning models were built to identify the patterns of changes in blood indicators related to HF. The CBC (complete blood cell count (CBC) is a common blood test that provides information about the cellular components of the blood, including red blood cells, white blood cells, and platelets. Changes in CBC parameters have been observed in various cardiovascular diseases, and these changes reflect underlying pathophysiological processes (13–16). Therefore, monitoring changes in these indicators can provide an important reference for the early diagnosis and treatment of HF.

## 2 Materials and methods

This study was approved by the Ethics Committee of First Affiliated Hospital of Zhejiang Chinese Medical University (reference number 2024-KL-551-01). The subjects of the study were 226 patients (including 127 patients with HF) who received treatment at the Hubin Campus of Zhejiang Provincial Hospital of Traditional Chinese Medicine between May 1, 2024, and June 30, 2024, and 211 patients from the other two hospital districts were retrospectively analyzed. Patient data, including electronic medical records and laboratory test indicators, were obtained from a hospital information system. The inclusion criteria were as follows: (1) Availability of complete clinical data; (2) A definite diagnosis of HF; (3) An average age of 65–75 years. The exclusion criteria were as follows: (1) Other infectious diseases; (2) Concurrent malignancy or severe blood/immune system disease; and (3) Blood transfusion therapy in the last month.

Detailed patient clinical information and data including age, sex, complications, and routine peripheral blood status were collected. Routine blood examination data (white blood cell count, neutrophil count, lymphocyte count, monocyte count, red blood cell count, hemoglobin level, and platelet count) were also recorded. The following conventional blood-derived indicators were calculated: neutrophil-to-lymphocyte ratio (NLR), dNLR (derived neutrophil ratio (dNLR), monocyte-to-lymphocyte ratio (MLR), NMLR (sum of the neutrophil-to-monocyte-to-lymphocyte ratio (NMLR), SIRI (systemic inflammatory response index (SIRI), and SII (systemic immune inflammation index). The calculation is as follows: NLR [Neutrophil counts 10^9^/L/lymphocyte count (10^9^/L)], dNLR = neutrophil count (10^9^/L) (white blood cell count-lymphocyte count) (10^9^/L), MLR = monocyte count (10^9^/L)/lymphocyte count (10^9^/L), NMLR = (monocyte count + neutrophil count) (10^9^/L)/lymphocyte count (10^9^/L), SIRI = neutrophil count (10^9^/L) Monocyte count (10^9^/L)/lymphocyte count (10^9^/L), SII = platelet count (10^9^/L) Neutrophil count (10^9^/L)/lymphocyte count (10^9^/L). Based on the examination results of the patient, the group of 226 patients were divided into two groups: a HF group and a normal control group.

The Beckman Couldt DxAI platform (https://www.xsmartanalysis.com/login/) was used for statistical analysis. Minimum Absolute contraction and selection operator (LASSO) regression analyses were used to identify the factors associated with HF. XGBoost, Logistic Regression (LR), LightGBM (LGBM), Random Forest (RF), AdaBoost, Decision Tree (DT), Gradient Boosting Decision Tree (GBDT), and Gaussian Naive Bayes (GNB), were the eight candidate models which were evaluated based on calibration plots and assessed for their predictive performance in terms of sensitivity, specificity, accuracy, predictive value, and area under the curve (AUC) in both test and validation queues. Random draw of 15.00% of the data in the overall sample as the test queues and the remaining samples as the training queues for 2-fold cross-validation (One served as training queues and one as validation queues) and tested in AUC = 0.9310 ± 0.0009 in the validation queues. The final model had an AUC = 0.8889 and an accuracy = 0.7647 in the test queues. This identified the optimal machine learning model. The filtered models were subsequently validated in an external independent test cohort.

SPSS Modeler 16.0 and the R software version 4.2.3 were used in this study. For measurement data, the *t*-test and Wilcoxon signed-rank test were used for analysis, and for count data, the chi-square test was used for comparison. The *t*-test was used for numerical variables with a normal distribution and homogeneity of variance, and the Wilcoxon signed-rank test was used for numerical variables with a normal distribution but uneven variance. LASSO regression analysis was used to identify factors associated with the development of HF and to assess the performance of these factors using receiver operating characteristic (ROC) curves. The statistical significance level was set at *P* < 0.05.

## 3 Results

### 3.1 Baseline characteristics

Table 1 presents the baseline characteristics of 226 patients. Among these patients, 127 (56.19%) were diagnosed with HF, of which 52 (40.95%) were female patients and 75 (59.05%) were male patients. The remaining 99 (43.81%) patients were healthy of which 49 (49.5%) were female and 50 (50.50%) were male. In this study, sex did not show a significant difference (*P* = 0.200). All other factors (Table 1) were significantly different between the groups.

**Table 1:** Baseline characteristics of the two groups of patients.

Characteristics		Normal control (*n* = 99)	Heart failure group (*n* = 127)	*P*-value
Sex, *n* (%)	Female	49 (49.495%)	52 (40.945%)	0.200
	Male	50 (50.505%)	75 (59.055%)	
Variable category				
WBC, median [IQR]		5.700 [5.000, 6.600]	6.500 [5.000, 8.200]	0.003
NE, median [IQR]		3.100 [2.500, 3.800]	4.300 [3.200, 6.300]	<0.001
LY, mean (±SD)		1.940 ± 0.444	1.256 ± 0.525	<0.001
MO, median [IQR]		0.400 [0.400, 0.500]	0.500 [0.400, 0.600]	0.027
RBC, median [IQR]		4.550 [4.300, 4.930]	3.670 [3.130, 4.090]	<0.001
HGB, mean (±SD)		138.980 ± 13.151	109.291 ± 23.368	<0.001
PLT, median [IQR]		232.000 [184.000, 260.000]	179.000 [135.000, 239.000]	<0.001
NLR, median [IQR]		1.607 [1.294, 1.957]	3.625 [2.400, 6.250]	<0.001
DNLR, median [IQR]		0.844 [0.806, 0.868]	0.878 [0.833, 0.908]	<0.001
MLR, median [IQR]		0.222 [0.182, 0.267]	0.400 [0.308, 0.556]	<0.001
NMLR, median [IQR]		1.867 [1.500, 2.176]	4.143 [2.667, 6.889]	<0.001
SIRI, median [IQR]		0.720 [0.500, 0.978]	1.733 [0.921, 3.300]	<0.001
SII, median [IQR]		375.158 [270.667, 480.000]	696.000 [373.333, 1,195.000]	<0.001

### 3.2 Feature selection related to HF

**Figure 1:**
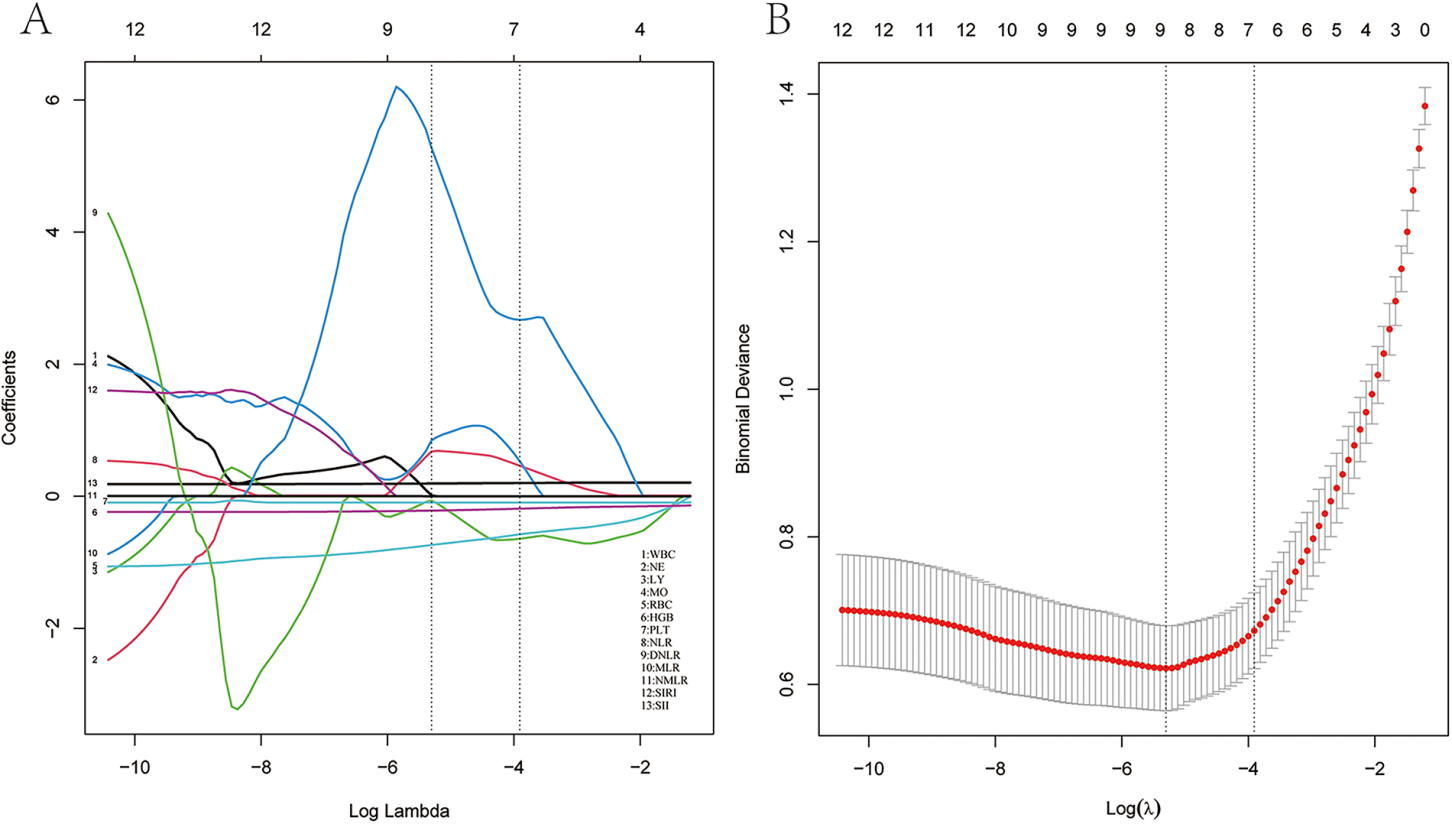
LASSO regression analysis and 10-fold cross-validation of risk factors associated with HF. **(A)** Nine non-zero coefficient risk factors were identified using the LASSO method. **(B)** Coefficient plot of generated log (*λ*) sequence.

**Figure 2:**
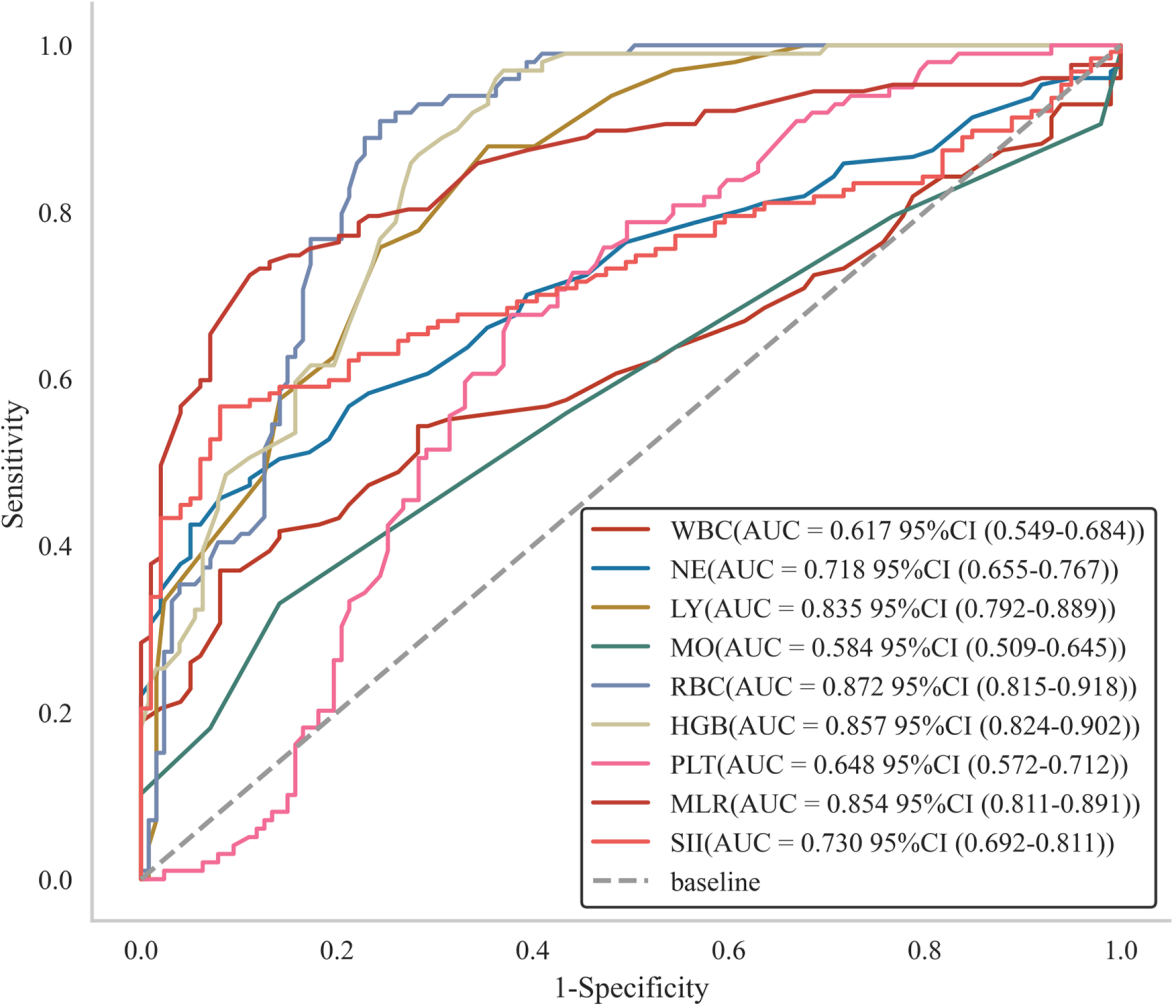
Subject operating characteristic (ROC) curves with different factors predicting the occurrence of HF.

LASSO regression analysis was performed to identify factors associated with the risk of HF (Table 1). The analysis showed that white blood cell count (WBC), neutrophil count (NE), lymphocyte count (LY), monocyte count (MO), red blood cell count (RBC), hemoglobin (HGB), platelet count (PLT), monocyte-to-lymphocyte ratio (MLR), and systemic inflammation index (SII) were risk factors for HF (see Figure 1). In addition, we assessed the AUC values of these factors by ROC analysis (Figure 2). The AUC values for WBC, NE, LY, MO, RBC, HGB, PLT, MLR, and SII, were 0.617, 0.718, 0.835, 0.584, 0.872, 0.857, 0.648, 0.854, and 0.730.

### 3.3 ML algorithm feature recognition

**Figure 3:**
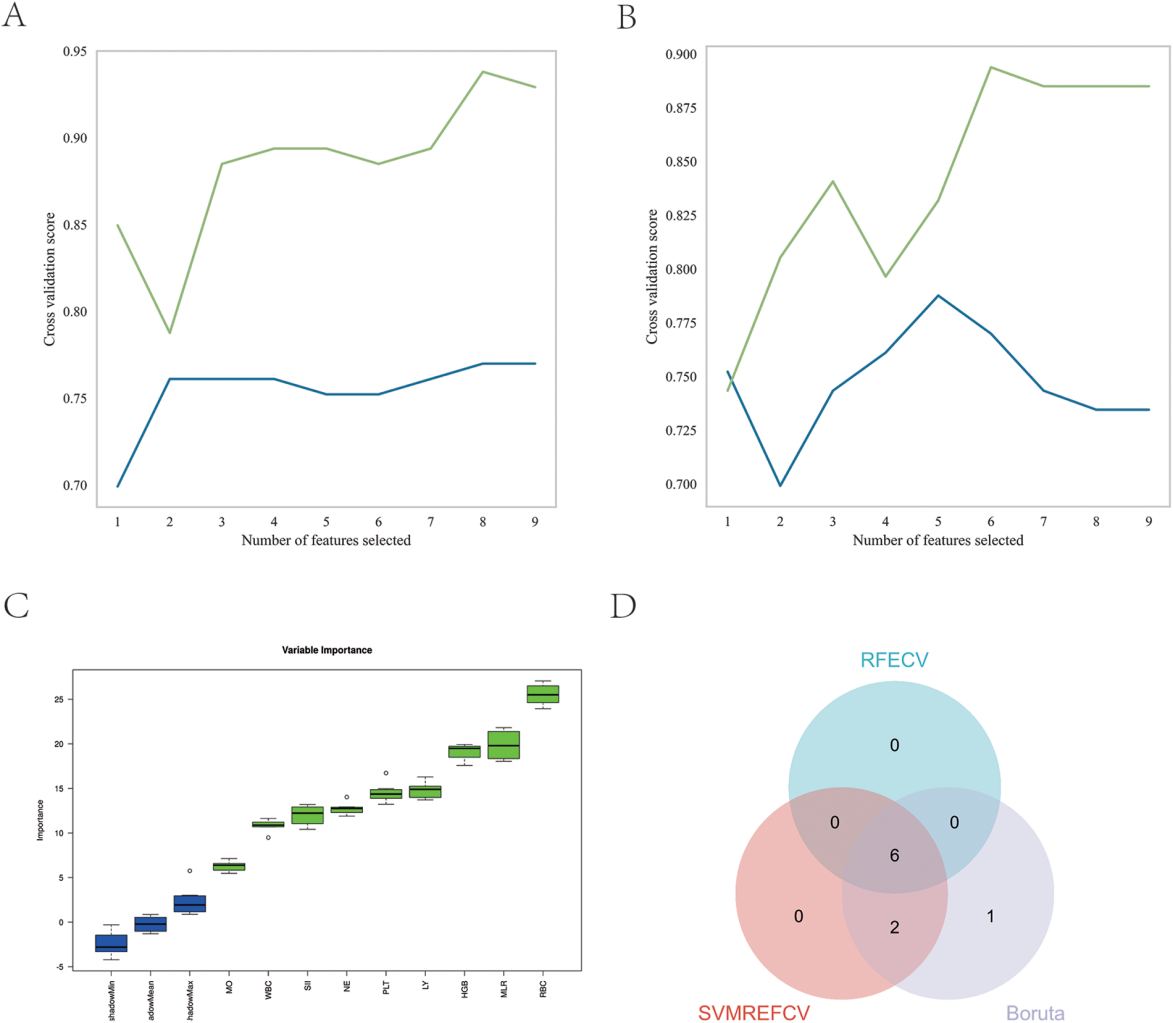
Feature screening. **(A)** Eight factors were selected using the SVMREFCV method; **(B)** six factors were selected using the RFECV method; **(C)** nine factors were selected using the Boruta method; **(D)** Venn diagram of the three machine learning algorithms.

The REFCV, SVMREFCV, and Boruta algorithms were used to identify markers (Figures 3A–C), and a Venn diagram was drawn using the R language (Figure 3D). Considering the intersections of the three algorithms, six overlapping markers were identified: WBC, NE, RBC, HGB, PLT, and MLR.

### 3.4 Optimal model identification

**Figure 4:**
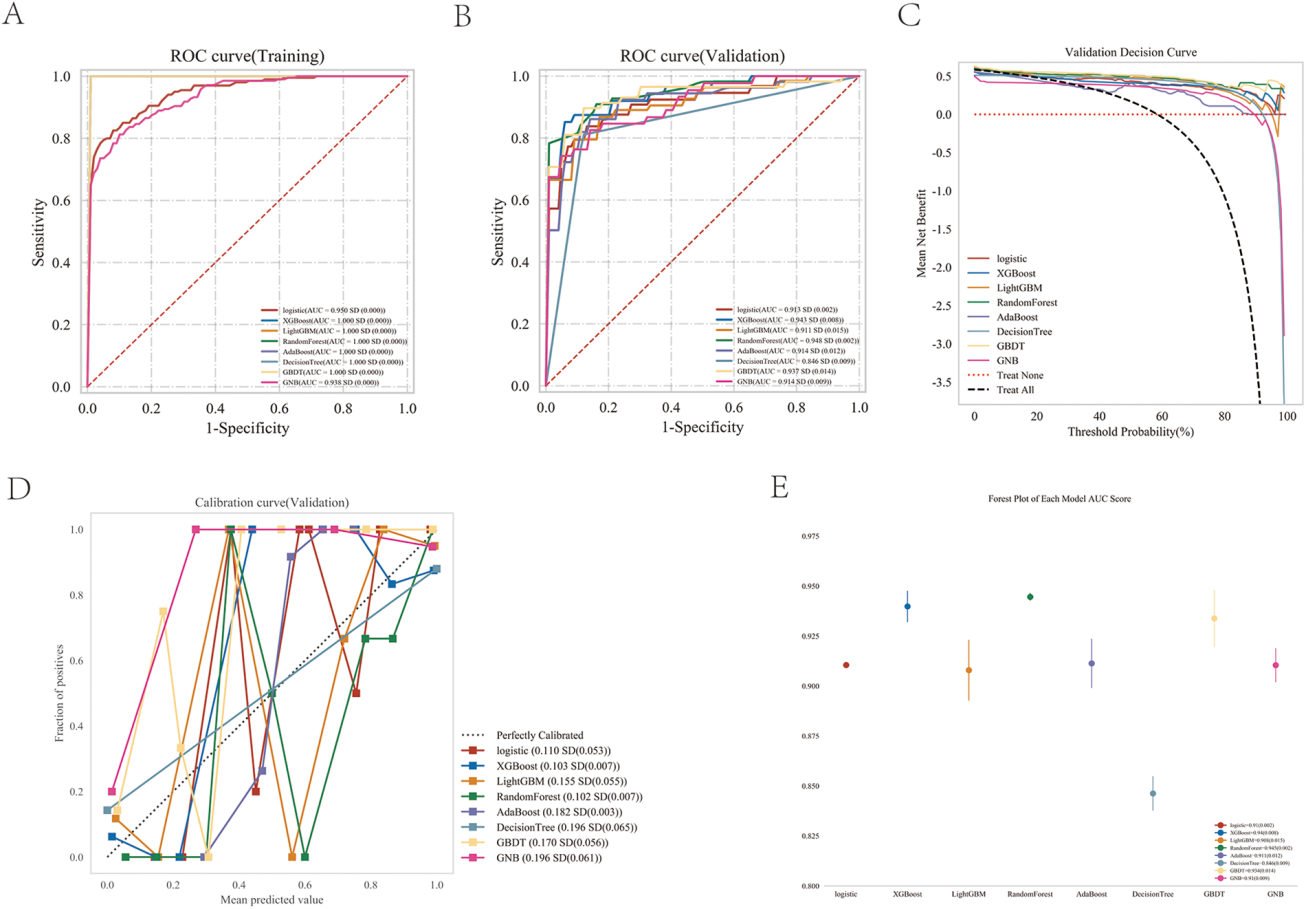
Performance comparison between multiple models. **(A)** ROC curve for the test cohort; **(B)** ROC curve for the validation cohort; **(C)** decision curve of the machine learning model; **(D)** calibration curve for the specific machine learning model; **(E)** forest area (AUC) in each area under the curve.

The performances of the eight machine learning models in the training and validation stages are presented in Table 2 and Figure 4. The random forest (RF) model was the most prominent in its prediction accuracy, with AUC values of 0.948 and 1.000 in the validation and testing stages, respectively. In addition, both the calibration and decision curves confirmed the excellent performance of the random forest (RF) model and its value in clinical applications.

**Table 2:** The diagnostic effect of the eight classification models in the training and validation cohorts.

Classifier	Cohorts	AUC	Cutoff	Accuracy	Sensitivity	Specificity	Positive predictive value	Negative predictive value	F1
Logistic	Training	0.945	0.671	0.858	0.769	0.974	0.975	0.766	0.860
	Validation	0.927	0.671	0.783	0.681	0.923	0.916	0.699	0.775
XGBoost	Training	1.000	0.771	0.994	0.990	1.000	1.000	0.987	0.995
	Validation	0.934	0.771	0.837	0.780	0.907	0.908	0.776	0.839
LightGBM	Training	1.000	0.757	0.994	0.990	1.000	1.000	0.988	0.995
	Validation	0.899	0.757	0.783	0.730	0.859	0.886	0.688	0.800
RandomForest	Training	1.000	0.525	0.992	0.990	0.994	0.995	0.987	0.993
	Validation	0.948	0.525	0.837	0.822	0.851	0.886	0.801	0.848
AdaBoost	Training	1.000	0.501	0.994	0.990	1.000	1.000	0.988	0.995
	Validation	0.920	0.501	0.859	0.826	0.930	0.963	0.708	0.889
DecisionTree	Training	1.000	1.000	0.450	0.000	1.000	NaN	0.450	NaN
	Validation	0.803	1.000	0.391	0.000	1.000	NaN	0.391	NaN
GBDT	Training	1.000	0.866	0.994	0.990	1.000	1.000	0.987	0.995
	Validation	0.908	0.866	0.783	0.683	0.920	0.889	0.695	0.771
GNB	Training	0.941	0.155	0.864	0.831	0.906	0.918	0.809	0.872
	Validation	0.928	0.155	0.793	0.686	0.948	0.947	0.693	0.790

### 3.5 Analysis of the random forest (RF) model

**Figure 5:**
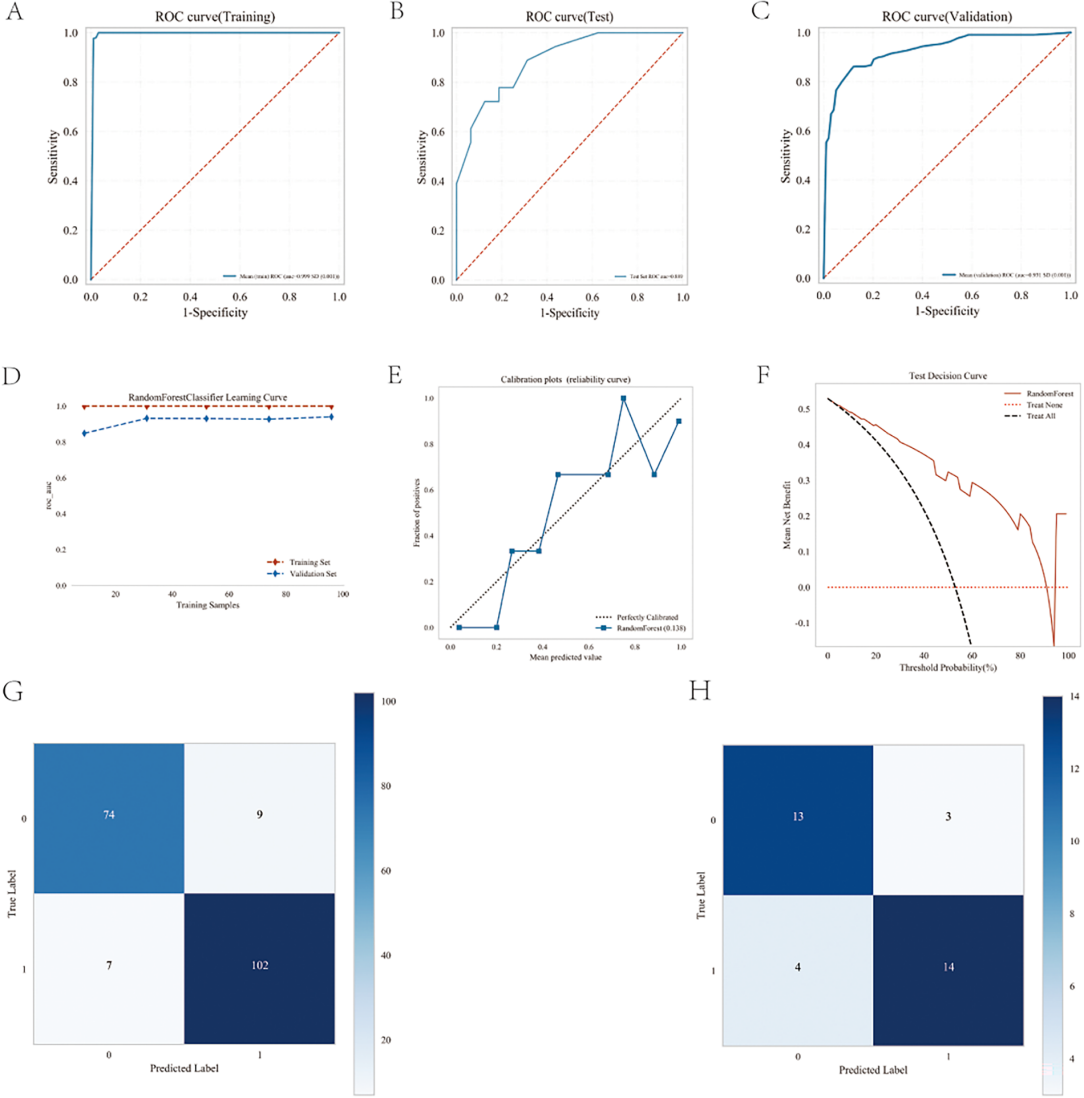
Performance of the predicted modes. **(A)** ROC curve for the test cohort; **(B)** ROC curve for the test cohort; **(C)** ROC curve for the validation cohort; **(D)** AUC for the test cohort; **(E)** calibration curve analysis; **(F)** decision curve analysis; **(G)** confounding matrix for the training set; **(H)** confounding matrix for the test set.

**Figure 6:**
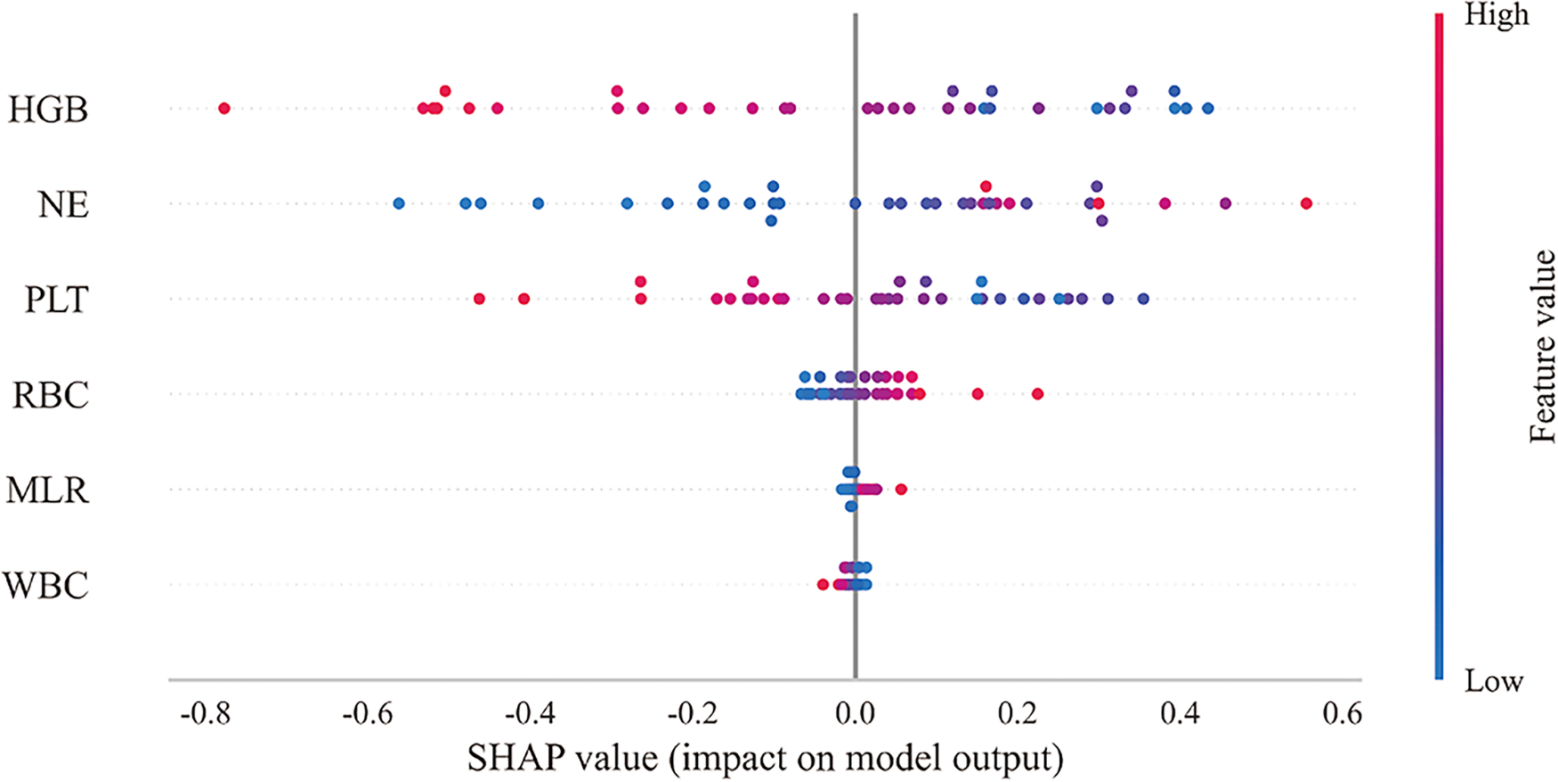
Overall SHAP explanations. SHAP explanations, red color represents higher values for covariates, while blue represents lower values for covariates. The *x*-axis represents the change in the log probability of having a heart failure.

As shown in Table 3 and Figure 5A–C, the AUC values of the test cohort were similar to the AUC values of the validation cohort, and the validation cohort did not surpass the training cohort in Figure 5D, indicating a strong fit of the random forest (RF) model. Table 3 shows that accuracy, sensitivity, and specificity exceeded the 70% threshold for the test sequence. Furthermore, the calibration curve demonstrated a significant correlation between the actual and predicted probabilities (Figure 5E), while the decision curve demonstrated the significant clinical utility of the model (Figure 5F), thus confirming the superior performance of the Random Forest (RF) model. The results of the confusion matrix show the performance of the model differences on different datasets. In the training set (Figure 5G), the true positive rate (sensitivity) was 93.6% and the true negative rate (specificity) was 89.2%. In the test set (Figure 5H), the true positive rate was 77.8% and the true negative rate was 81.3%. Figure 6 shows the overall SHAP interpretation for all model covariates applicable to predict the probability of occurrence of heart failure.

**Table 3:** Diagnosis effect of random forest (RF) models in the test and validation cohort.

Cohorts	AUC	Cutoff	Accuracy	Sensitivity	Specificity	Positive predictive value	Negative predictive value	F1
Training	0.999	0.600	0.974	0.963	0.988	0.991	0.953	0.977
Validation	0.931	0.600	0.844	0.788	0.915	0.928	0.776	0.849
Testing	0.889	0.550	0.754	0.722	0.813	0.813	0.722	0.765

### 3.6 External validation of the random forest (Rf) model

**Figure 7:**
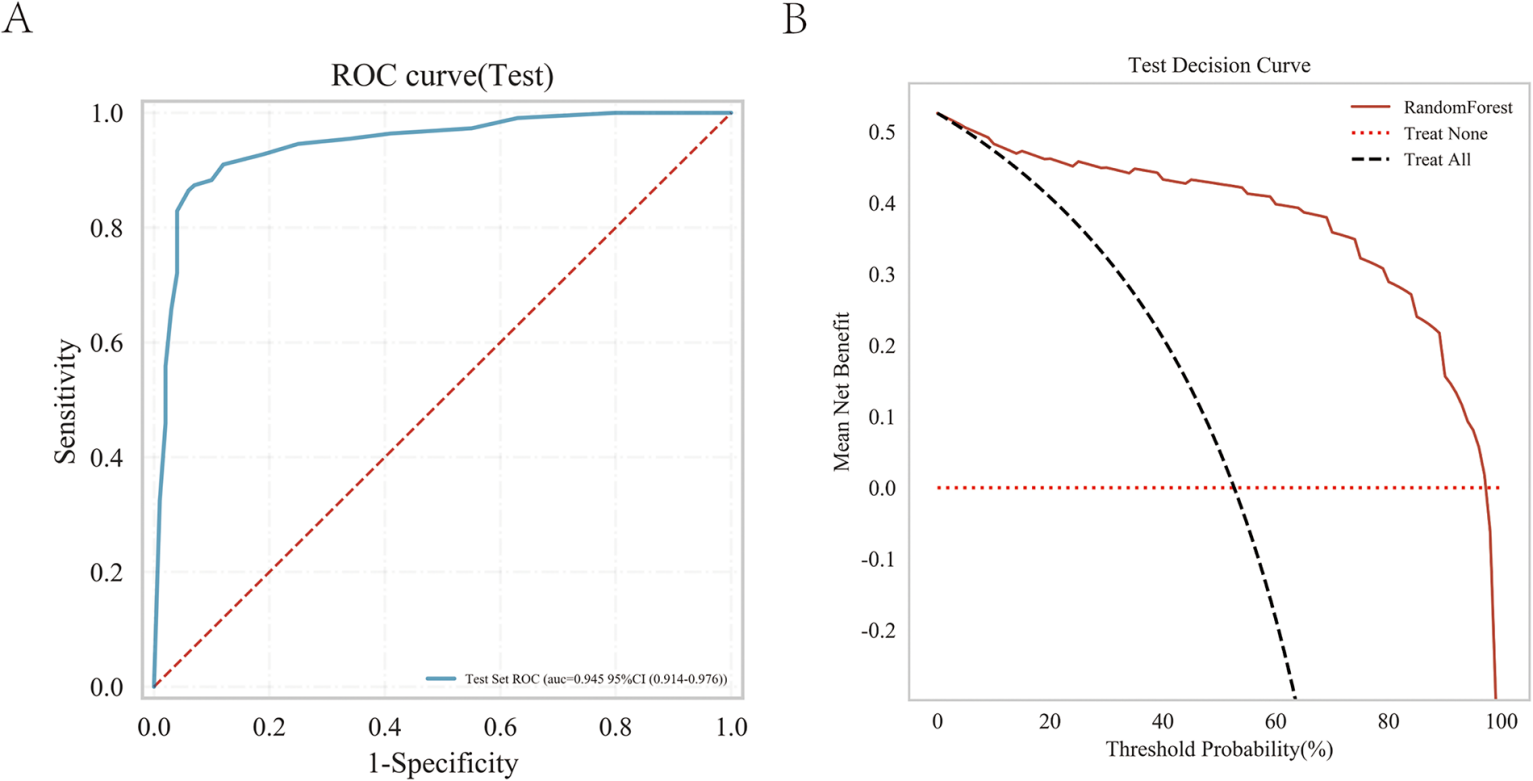
External independent test of the random forest (RF) model. **(A)** Subject operating characteristics (ROC) curves for the external independent testing cohort. **(B)** Test decision curve for the external independent test cohort.

An external independent test cohort of 211 patients from the other two districts. The area under the curve (AUC) is 0.945 (Figure 7A), and the decision curve revealed a significant clinical benefit (Figure 7B).

### 3.7 Online forecast site

**Figure 8:**
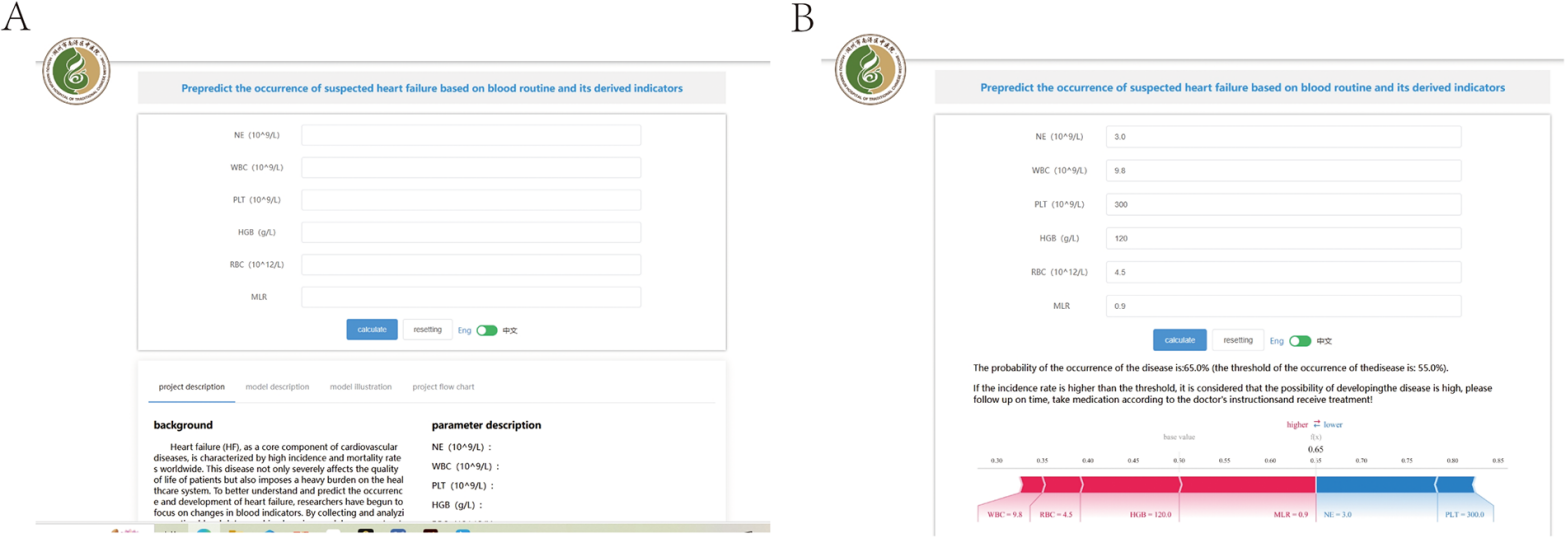
An online prediction tool **(A,B)** that predicts the probability of developing heart failure based on the random forest (RF) model, according to the 6An online page for several indicators to predict risk.

Following the above analysis, we developed an online prediction tool designed to help primary clinicians assess the risk of HF occurrence in patients with suspected HF. This tool allows users to input blood indicators (WBC, NE, RBC, HGB, PLT, and MLR) to predict the likelihood of disease occurrence (Figure 8). If the results indicate a high risk of death, clinicians should be vigilant and prepare for treatment in advance. (http://www.xsmartanalysis.com/model/list/predict/model/html?mid=18904&symbol=71aW7309uF6WP3170Zn1).

## 4 Discussion

In HF patients, a decline in the heart's ability to pump blood leads to an increased cardiac workload, which, in turn, prompts cardiac muscle cells to secrete more pro-BNP. Therefore, pro-BNP levels can serve as a key indicator for assessing cardiac function (17). However, despite the important clinical value of the pro-BNP test in the diagnosis of HF, it can help physicians quickly identify and evaluate the severity of HF, thus providing timely and effective treatment. However, in primary medical institutions, the popularization and application of pro-BNP testing still faces certain challenges owing to equipment and technical limitations. Many primary hospitals lack advanced testing equipment to perform accurate pro-BNP testing and the professional level of technicians not standardized, further limiting the wide application of pro-BNP testing in primary medical institutions. Therefore, identifying new early diagnostic markers is of great importance for improving the prognosis of patients with HF, improving their quality of life, and reducing the burden on the medical system. Moreover, by reducing the burden on the medical system, the allocation of medical resources can be optimized such that more patients can obtain timely and effective treatment, thereby improving the overall health of society.

With the widespread adoption of electronic health records, hospital information systems have accumulated abundant patient visit data worldwide, creating an ideal environment for machine learning (ML) applications. Traditional regression analysis methods have limitations in handling complex high-dimensional interaction information in large datasets, technically limiting the ability of models to make effective predictions regarding complex relationships. Machine learning can effectively overcome these challenges (18). When processing complex data, machine learning (ML) does not need to preset the nature of the data distribution or the linear or nonlinear connections between features. Machine learning can assist in identifying potential predictor variables and models using computationally intensive iterative algorithms rather than relying on manually selected features, thus improving the prediction accuracy of the model (19). In recent years, machine-learning technology has been widely used in cardiovascular medicine, particularly in the management of HF patients. For example, machine learning techniques have been used in the diagnosis of HF, death risk prediction, and evaluation of rehospitalization rates, and have demonstrated excellent efficacy (20, 21). The use of random forest models can accurately predict the risk of heart failure in middle-aged and elderly individuals in pre-diabetes or diabetic states (22). The random forest algorithm utilizes the a reliable method for a combined model of individual features to improve the accuracy of high-frequency predictions (23). Studies have demonstrated the ability of the random forest model to identify risk factors in patients with HF (24). To enhance clinical physicians' recognition and trust in machine learning models, this study drew on the research findings of Alexander A. Huang et al. (25, 26) and introduced the SHAP value analysis method. This method, by enhancing the transparency of the model, further strengthens the model's credibility and the practicality of its clinical application.

Routine blood testing contains a lot of data on the disease, and is more economical and acceptable to patients than pro-BNP. By collecting and analyzing routine blood data, machine learning models were designed to identify the patterns of changes in blood indicators related to HF. This method adopts advanced data analysis technology, which can screen valuable information from a large number of blood indicators and provide a scientific basis for the early diagnosis and treatment of HF. Using this method, doctors can more accurately assess a patient's condition and develop targeted treatment plans to improve the treatment effect and reduce the morbidity and mortality. Therefore, after evaluating eight machine learning models, we developed a prediction model that used laboratory data to predict the risk factors associated with the occurrence of HF.

This study demonstrated significant associations between six blood markers (white blood cell count, neutrophil count, red blood cell count, hemoglobin level, platelet count, and monocyte-lymphocyte ratio) and the occurrence of HF. Among the eight machine learning algorithms evaluated, the Random Forest (RF) model showed the highest prediction accuracy and achieved high AUC values in both the validation and test sets. This result is consistent with Chicco D et al. (24).

Leukocytes constitute a key part of the immune system, and their main role is to resist infection and participate in inflammatory responses. HF manifests as a decline in heart function, making the heart unable to efficiently pump blood to meet its needs (27). The association between white blood count and HF is mainly reflected in the inflammatory response. Studies have found that damage due to HF can lead to the release of inflammatory mediators, such as interleukins (ILs) and tumor necrosis factor (TNF), which stimulate the bone marrow to release more white blood cells into circulation, leading to an increase in the white blood cell count. Furthermore, according to relevant research findings, there is a strong association between a higher neutrophil count and HF, even within the normal range. Specifically, the comparison between neutrophil counts of 6–7  ×  10^9^/L vs. 2–3 × 10^9^/L shows a strong correlation with HF (HR: 2.04; 95% CI: 1.82–2.06). This suggests that the white blood cell count, especially the level of neutrophils, may be associated with an increased risk of HF (28) therefore, the analysis of the white blood cell count and subtypes may be very valuable in assessing the inflammatory status and prognosis of patients with HF. In this study, white blood cell counts were generally higher in patients with HF than in healthy controls, and the results were consistent with the findings of previous studies. An increase in the white blood cell count can be used as an indicator of inflammation in HF to assess disease severity and prognosis.

As primary oxygen-carrying cells, a reduction in the number of red blood cells leads to a decrease in the ability of blood to transport oxygen, which can exacerbate myocardial ischemia and hypoxia. To compensate for this deficiency, the heart attempts to increase blood supply by accelerating the heart rate and enhancing myocardial contractility. However, this also increases the workload on the heart. Long-term reduction in red blood cells, along with myocardial ischemia and hypoxia, can lead to gradual deterioration of heart function. To meet the body's oxygen demand, the heart must exert more force to pump blood. This persistent increase in cardiac workload may lead to a gradual enlargement of the heart, weakened myocardial contractility, and ultimately result in the occurrence of HF (29). This is also highly consistent with the results of this study, further validating our findings and conclusions.

Platelets are also involved in blood flow. When the number of thrombocytes is reduced, the heart requires more power to maintain blood circulation, thereby increasing the burden on the heart. Long-term burden may lead to gradual impairment of cardiac function, leading HF. Studies have found that thrombocytopenia is a common platelet abnormality in patients with HF (30). In this study, the platelet count in the experimental group was lower than that in the control group, which may have been because the majority of the patients were taking anticoagulant drugs, resulting in reduced platelet levels.

Our study has certain limitations. First, the sample size was relatively small. Second, the data were derived from a single center and were retrospective. Moreover, the population we selected is the elderly population, Therefore, this model has an accurate predictive power for the probability of developing heart failure in the elderly population. In the future, we will gradually expand the sample size and include more covariates, as well as broaden the scope of the model, based on the progress of our research and the availability of resources and we will consider combining this model with additional clinical and imaging data to enhance the predictive power of the model.

## 5 Conclusions

This study identified key factors associated with the occurrence of HF using the LASSO regression method, including white blood cell count (WBC), neutrophil count (NE), red blood cell count (RBC), hemoglobin level (HGB), platelet count (PLT), and monocyte to the lymphocyte ratio (MLR). After evaluating eight different machine learning models, the Random Forest (RF) model was found to perform the best, demonstrating excellent predictive accuracy and clinical utility. A significant positive correlation was observed between the probability of actual occurrence and the probability predicted by the model. In addition, the online prediction tool can effectively assist doctors in primary medical institutions to predict a patient's condition more accurately.

## Data Availability

The raw data supporting the conclusions of this article will be made available by the authors, without undue reservation.
